# Evaluation of Negative Pressure Wound Therapy in the Management of Fournier’s Gangrene

**DOI:** 10.7759/cureus.48300

**Published:** 2023-11-05

**Authors:** Sushant Tanwar, Shivani B Paruthy, Arun Singh, Vikas Pandurangappa, Deepak Kumar, Soni Pal

**Affiliations:** 1 Department of General Surgery, Vardhman Mahavir Medical College and Safdarjung Hospital, New Delhi, IND; 2 Department of General Surgery, Safdarjung Hospital, New Delhi, IND

**Keywords:** fournier's gangrene, negative pressure wound therapy, vacuum-assisted closure (vac), conventional dressing, ­wound healing

## Abstract

Introduction

Necrotizing soft tissue infections (NSTIs), including Fournier's gangrene (FG), are severe polymicrobial bacterial infections characterized by rapidly spreading inflammation and tissue necrosis. This study aims to compare the clinical outcomes of vacuum-assisted closure (VAC) dressing and conventional dressing in patients with FG.

Materials and methods

A prospective study was conducted from December 2020 to May 2022, including patients with clinical features suggestive of FG. Patients were divided into two groups: conventional dressing and VAC dressing. Relevant clinical data, including age, duration of hospital stay, wound status, Fournier's gangrene severity index (FGSI) scores, sepsis markers (C-reactive protein (CRP), neutrophil-to-lymphocyte ratio, and procalcitonin), and pain assessment, were recorded and compared between the two groups.

Results

A total of 84 patients were included in the study, with 42 patients in each group. The mean age was 57.48 ± 15.74 years in the conventional dressing group and 50.83 ± 13.95 years in the VAC dressing group. VAC dressing was associated with a significantly shorter duration of hospital stay (8.14 ± 3.13 days) compared to conventional dressing (11.36 ± 4.75 days). The average time taken for wound closure was significantly reduced in the VAC dressing group (63 ± 14.81 days) compared to the conventional dressing group (112.56 ± 13.82 days). FGSI scores showed significant improvement after debridement in both groups, with lower scores in the VAC dressing group at discharge. Sepsis markers such as CRP and serum procalcitonin exhibited a significant decrease after VAC application.

Discussion

The study demonstrates that VAC therapy is associated with better clinical outcomes in FG, including reduced duration of hospital stay, faster wound closure, improved FGSI scores, decreased sepsis markers, and reduced pain. These findings align with previous studies highlighting the advantages of VAC therapy over conventional dressing methods.

Conclusion

VAC therapy provides significant benefits in the management of FG, leading to improved clinical outcomes and patient quality of life. It offers advantages such as shorter hospital stays, faster wound closure, and reduced sepsis markers. The application of VAC dressing should be considered a valuable treatment modality for FG.

## Introduction

Necrotizing soft tissue infections (NSTIs) are polymicrobial bacterial infections characterized by rapidly spreading inflammation and necrosis of skin, subcutaneous tissue, and fascia, which include necrotizing forms of fasciitis, myositis, and cellulitis [1,2]. Hippocrates first described NSTI in the 5th century BC, as a complication of erysipelas [3]. Such an infective NSTI involving the genital, perineal, or perianal region was named Fournier's gangrene (FG). FG was first described by Sir Alfred Fournier in 1883; he stated it as an idiopathic process with a common source of infection. FG is a potentially fatal NSTI of external genitalia [4]. The infective process arises from the urogenital tract, anorectum, or cutaneous source and is associated with immunosuppression [5]. Early detection with surgical debridement along with aggressive fluid resuscitation and broad-spectrum antibiotics is mandatory for the management of this debilitating disease [6].

Repeated conventional dressing with aggressive surgical debridement is required in the initial period of treatment, which results in tissue defects that may take a long time to close [7].

Many surgeons worldwide have started using negative pressure wound therapy (NPWT) for fast and effective wound closure. NPWT creates an ideal wound healing environment through several putative mechanisms and it has been a breakthrough in such complex and difficult wounds by creating a faster wound bed granulation, which is a foundation for complete wound closure spontaneously or surgically [8,9].

A vacuum-assisted closure (VAC) device consists of a sterile, open-cell foam sponge that is placed in the wound, the size of which can be adjusted to the wound size. This is covered with a transparent adhesive drape to create an airtight environment. The sponge is connected to a portable vacuum pump using non-collapsible tubing. Evacuation is applied to the sponge using the pump or wall mount suction, which provides continuous regulated negative pressure. In VAC devices, wound healing is improved by providing macro and microstrain foam dressing under negative pressure, applying both mechanical and biological forces to the wound to create an environment that promotes wound healing. These forces are known as macrostrain and microstrain [10-12].

VAC device increases healing by providing microstrains that promote wound closure by fibroblast migration, cell mitosis, and proliferation, while the negative pressure decreases the edema and exudate collection.

VAC helps in the early closure of wounds but perineal VAC dressing is a challenge in itself and technically difficult because of the site and proximity of genitalia to the anal region. Infective organisms spread via Colles fascia in the perineum and via dartos fascia in the scrotum. The infection further advances through Scarpa’s fascia to the abdominal wall. After radical debridement of the wound in multiple sittings, it is difficult to leave the wound open. Healing by secondary intention requires prolonged hospitalization and reconstruction flaps are at risk of failure. Successful placement of vac dressing and subsequent changes every 4th day avoid urinary and fecal contamination, it also helps in giving a psychological boost to the patient.

FG is associated with infection of the genital, perineal, and abdominal regions. A mortality of 20% is reported within the initial phase if timely intervention is not undertaken.

Aerobic and anaerobic bacteria such as *Streptococci*, *Escherichia coli*, and *Staphylococcus* produce enormous amounts of collagenase, heparinase, and streptokinase. Super-infection with poly-microbial flora as gram-positive, gram-negative, and anaerobes contributes to the release of enzymes that destroy the tissue leading to obliterative endarteritis and thrombosis of vasculature of skin and subcutaneous tissue resulting in the spread of infection [13].

Besides aggressive treatment, FG is associated with high morbidity and mortality (3-67%) because of delay in diagnosis. A different treatment protocol has been proposed in the past for postoperative wound management, including unprocessed honey, repeated conventional dressing and debridement, hyperbaric oxygen, and VAC devices [14]. Conventional dressing and debridement lead to a wound that takes a long time to close and heal properly. VAC dressing besides helping in the wound care system also helps in removing exudates, reducing edema and contraction of the scar, and promoting wound healing, which is easy to manage.

Hence, to improve the outcome of FG, this study is undertaken using VAC dressing, which works on the basis of NPWT with ease of ambulation and early healing of the wound. Also, conventional dressing was reserved for those who refused to participate in VAC dressings.

## Materials and methods

For this study, institutional ethics committee approval was taken from Vardhman Mahavir Medical College and Safdarjung Hospital (IEC/VMMC/SJH/Thesis/2020-11/CC-146).

Sample size

Our estimated sample size was based on the study of the role of VAC therapy in the management of FG by Iacovelli et al. [14]. Thus sample size of 42 provided 80% power for detecting a significant difference between cohorts at an alpha level of 0.05. An equal number of patients were included in the conventional group who refused consent for VAC.

From December 2020 to May 2022, all the patients fulfilling the inclusion criteria with clinical features suggestive of FG (pain, erythema, swelling, crepitations, and ischemic changes of scrotal skin) were admitted to the department of general surgery and were included in the study.

A thorough history and clinical examination were done and associated comorbidities were noted. Hematological, biochemical, and radiological investigations were done and broad-spectrum antibiotics containing third-generation cephalosporin, aminoglycoside, and metronidazole or clindamycin were started.

Patients were subjected to thorough debridement under appropriate anesthesia and tissues were sent for histopathology and culture sensitivity. Associated comorbidities, including diabetes and deranged renal and hepatic functions, were taken care of as per requirement.

After the initial debridement of the wound, culture and sensitivity of the wound discharge were done. Patients negative for anaerobic growth were subjected to NPWT and consent for NPWT was taken prior to the procedure.

Patients with a positive anaerobic culture were not subjected to NPWT till a negative culture for anaerobes was received. Patients not giving consent for NPWT were subjected to conventional dressing (hydrogen peroxide and povidone-iodine) and all the sepsis scores and Fournier's gangrene severity index (FGSI) were observed as in the case of NPWT.

Negative vacuum pressure around 75-125 mmHg was created in these cases and the dressing was changed every 72 hours. Serial debridement was done as per requirement.

Markers such as procalcitonin, C-reactive protein (CRP), and neutrophil-to-lymphocyte (N:L) ratio were checked every fourth day. FGSI scoring was done at the time of admission, after the first dressing was changed, and at the time of discharge for each patient, which includes nine parameters, including temperature, heart rate, respiratory rate, sodium levels, potassium levels, creatinine, bicarbonate levels, hematocrit, and total leukocyte count. Each parameter is graded from 0 to +4 on either side of the normal value and summed up to obtain an FGSI score.

Patients' quality of life results after FG were evaluated, including pain assessment. Wound closure was assessed based on the length and depth of the wound. Length of hospital stay was also evaluated. Patients were evaluated till the closure of the wound in surgical OPD for follow-up.

## Results

A total of 84 patients aged >12 years with a diagnosis of FG were admitted during the period of study, out of which in 42 patients, conventional dressing was done, and in 42 patients, VAC dressing was applied after initial debridement in the Department of General Surgery, Vardhman Mahavir Medical College & Safdarjung Hospital, New Delhi.

Age distribution

The age distribution of the patients in our study ranged from 22 to 81 years with a mean age of 57.48 ± 15.74 in the conventional group and 50.83 ± 13.95 in the VAC dressing group, as shown in Table 1. The age-wise distribution between both groups is shown in Table 2.

**Table 1:** Comparison of mean age between both groups VAC: vacuum-assisted closure.

	Conventional group (n = 42)	VAC dressing group (n = 42)	P-value
Mean age in years	57.48 ± 15.74	50.83 ± 13.95	0.06

**Table 2:** Age-wise distribution between both groups VAC: vacuum-assisted closure.

Age group	Conventional group (n = 42)	VAC dressing group (n = 42)
Upto 30 years	3 (7.1%)	5 (11.9%)
31-45 years	5 (11.9%)	10 (23.8%)
45-60 years	18 (42.9%)	18 (42.9%)
61-75 years	10 (23.8%)	8 (19.0%)
>75 years	6 (14.3%)	1 (2.4%)

Duration of hospital stay

In our study, patients in whom VAC dressing was done were admitted to the hospital for 8.14 ± 3.13 days, whereas patients in whom conventional dressing was done were admitted for 11.36 ± 4.75 days, which has a statistically significant value of 0.001, as shown in Table 3.

**Table 3:** Comparison of duration of hospital stay between both groups VAC: vacuum-assisted closure.

	Conventional dressing (n = 42)	VAC dressing group (n = 42)	P-value
Duration of hospital stay	11.36 ± 4.75	8.14 ± 3.13	0.001

Status of the wound

The average time taken for wound closure of patients in whom conventional dressing was done was 112.56 ± 13.82 days and patients in whom VAC dressing was applied achieved wound closure in 63 ± 14.81 average number of days, with a statistically significant p-value of <0.001, as shown in Table 4.

**Table 4:** Comparison of the number of days taken for wound closure between both groups VAC: vacuum-assisted closure.

	Conventional group (n = 42)	VAC dressing group (n = 42)	P-value
No. of days for wound closure	112.56 ± 13.82	63.0 ± 14.81	<0.001

Fournier’s gangrene severity index

In our study, at the time of admission, patients in whom VAC was applied had an FGSI average score of 6, and patients in whom conventional dressing was done had an average score of 7. However, after initial debridement, the VAC dressing group had an average score of 2, and the conventional dressing group had an average score of 3, with a statistically significant p-value of 0.02. At the time of discharge, the conventional group had an average FGSI value of 2 whereas the VAC group had an average value of 0, which leads to a statistically significant p-value of 0.001, as shown in Table 5.

**Table 5:** Comparison of FGSI score between both groups at different intervals FGSI: Fournier's gangrene severity index; VAC: vacuum-assisted closure.

FGSI	Conventional group (n = 42)	VAC dressing group (n = 42)	P-value
At admission	7 (4.75-9)	6 (3-10.25)	0.70
After debridement	10 (9-11)	7 (4-10.25)	<0.001
After dressing	3 (3-5)	2 (0-5)	0.02
At the time of discharge	2 (0-2)	0 (0-0)	0.001

Sepsis markers

A comparison of sepsis markers was done on the basis of CRP levels, N:L ratio, procalcitonin levels, and serum albumin levels.

CRP Level

At the time of admission, the average CRP value of the conventional group was 200 mg/l, and the average value of the VAC group was 150 mg/l. After the initial debridement, the group in which conventional dressing was done had an average CRP value of 115 mg/l, and the average value of the VAC group was 98 mg/l, which shows a statistically significant p-value of 0.001. At the time of discharge, the conventional dressing group had an average CRP value of 70 mg/l, and the average CRP value of the VAC group was 86 mg/l, which again shows a statistically significant p-value of 0.004, as depicted in Table 6.

**Table 6:** Comparison of CRP levels between both groups at different intervals CRP: C-reactive protein; VAC: vacuum-assisted closure.

CRP level	Conventional group (n = 42)	VAC dressing group (n = 42)	P-value
At admission	200 (160-240)	150 (110-215)	0.19
After debridement	166 (146-200)	230 (138-254.5)	0.003
After dressing	115 (100-140.5)	98 (90-109.25)	0.001
At the time of discharge	70 (60-80)	86 (64.25-89.25)	0.004

N:L Ratio

At the time of admission, the average N:L ratio of the conventional dressing group was 6.05, and of the VAC dressing group was 6.02. After debridement, for patients in whom conventional dressing was done, the average N:L ratio was 6.4, and for patients in whom VAC dressing was applied, the average N:L ratio was 7, which had a p-value of 0.54, which is not statistically significant. At the time of discharge, the average N:L ratio in the conventional dressing group was 6.8, and in the VAC dressing group, it was 6.55, which has a p-value of 0.96, which is not statistically significant, as depicted in Table 7.

**Table 7:** Comparison of N:L ratio between both groups at different intervals N:L ratio: neutrophil-to-lymphocyte ratio; VAC: vacuum-assisted closure.

N:L ratio	Conventional group (n = 42)	VAC dressing group (n = 42)	P-value
At admission	6.05 (3.22-9.37)	6.02 (3.22-8.49)	0.93
After debridement	10.2 (6.75-13.72)	11.45 (7.05-14.2)	0.37
After dressing	6.4 (5.4-8.4)	7 (5.5-8.4)	0.54
At the time of discharge	6.8 (5.4-8.45)	6.55 (5.55-8.8)	0.96

Procalcitonin Levels

At the time of admission, patients in whom conventional dressing was applied had an average value of serum procalcitonin of 0.32 ng/l, and in patients in whom VAC dressing was applied, the average value of procalcitonin was 0.30 ng/l. After debridement, patients in whom conventional dressing was done had an average value of 0.23 ng/l of serum procalcitonin and patients in whom VAC dressing was applied had an average value of 0.22 ng/l, with a p-value of 0.80, which is not statistically significant. At the time of discharge, the average value of serum procalcitonin in the conventional dressing group was 0.20 ng/l, and the average value of serum procalcitonin in the VAC group at the time of discharge was 0.20 ng/l, but had a statistically significant p-value of 0.004, as depicted in Table 8.

**Table 8:** Comparison of serum procalcitonin levels between both groups at different intervals VAC: vacuum-assisted closure.

Procalcitonin	Conventional group (n = 42)	VAC dressing group (n = 42)	P-value
At admission	0.32 (0.28-0.36)	0.30 (0.24-0.33)	0.04
After debridement	0.26 (0.20-0.30)	0.23 (0.20-0.30)	0.67
After dressing	0.23 (0.21-0.24)	0.22 (0.21-0.24)	0.80
At the time of discharge	0.20 (0.20-0.22)	0.20 (0.10-0.20)	0.004

Serum Albumin Levels

At the time of admission, the patients with conventional dressing had an average value of serum albumin of 2.60 g/l, and patients in whom VAC dressing was used had an average value of 2.10 g/l. After initial debridement, conventional dressing patients had an average value of 2.5 g/l of serum albumin, and patients in whom VAC dressing was applied had an average value of 3.05 g/l, with a statistically significant p-value of <0.001. This signifies that there is less loss of serum albumin in cases of VAC application, which promotes better wound healing, as depicted in Table 9.

**Table 9:** Comparison of serum albumin levels between both groups at different intervals VAC: vacuum-assisted closure.

Serum albumin	Conventional group (n = 42)	VAC dressing group (n = 42)	P-value
At admission	2.60 (2.07-2.80)	2.10 (1.80-2.4)	0.005
After debridement	2.8 (2.4-3.2)	2.30 (1.97-2.6)	<0.001
After dressing	3.05 (2.80-3.20)	2.5 (2-2.7)	<0.001
At the time of discharge	3.2 (3-3.2)	3.2 (3-3.3)	0.50

Pain assessment

At the time of admission, the average pain assessment score (done on the basis of the numeric pain rating scale) in patients with conventional dressing was 9 and the group in which VAC dressing was applied was 8. However, after initial debridement, conventional dressing patients' average pain assessment score after dressing was 5.5, and in the group in which VAC dressing was done, the average pain assessment score was 3, with a statistically significant p-value of <0.001, as shown in Table 10.

**Table 10:** Comparison of pain assessment scores between both groups at different intervals VAC: vacuum-assisted closure.

Pain assessment score	Conventional group (n = 42)	VAC dressing group (n = 42)	P-value
At admission	9 (8-9)	8 (7-9)	0.002
After debridement	9 (8-9)	10 (8-10)	<0.001
After dressing	5.5 (4.5-6)	3 (3-4)	<0.001
At the time of discharge	2 (2-2)	2 (2-2)	0.10

Image collection

**Figure 1:**
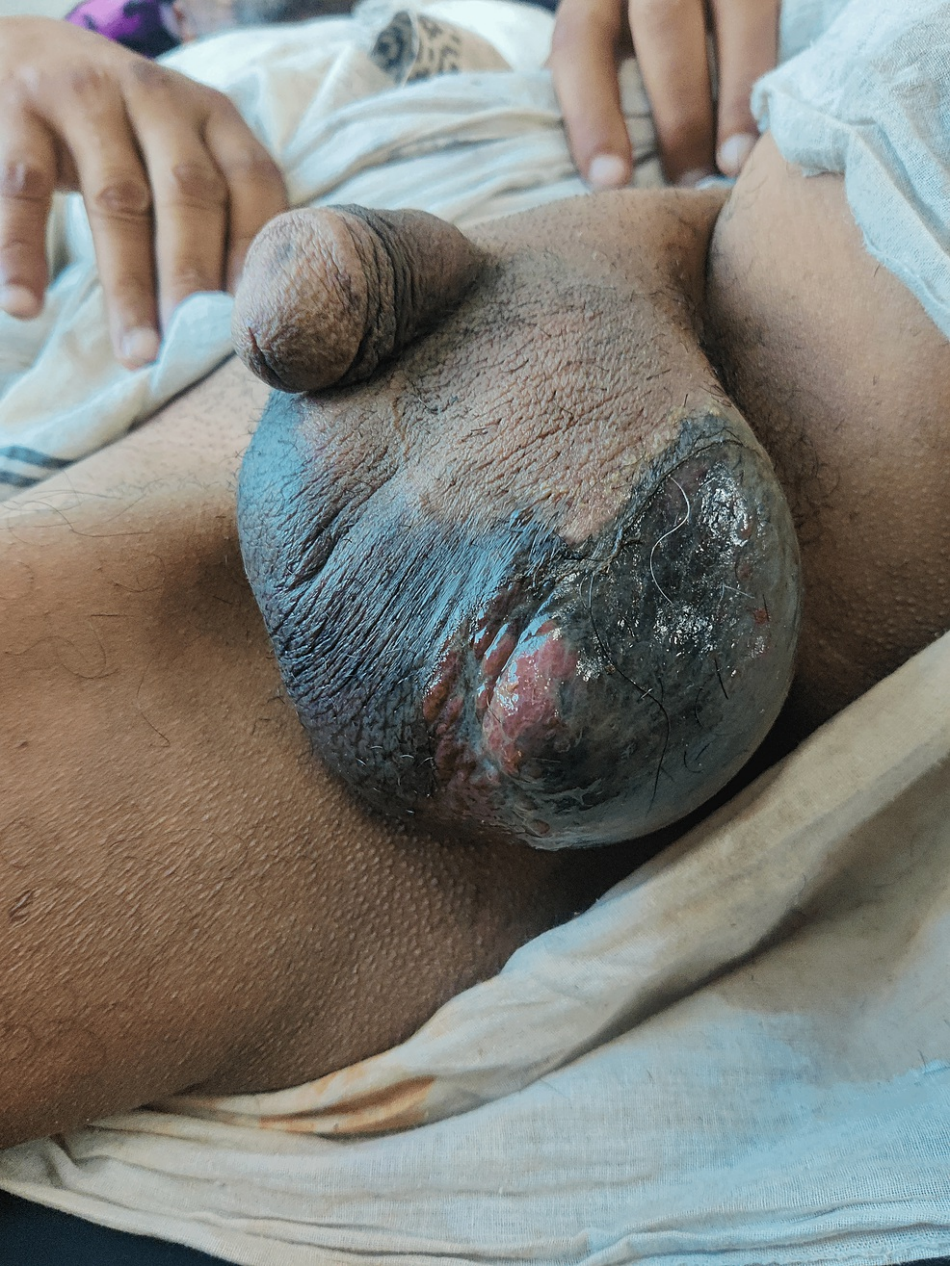
Fournier's gangrene at the time of presentation

**Figure 2:**
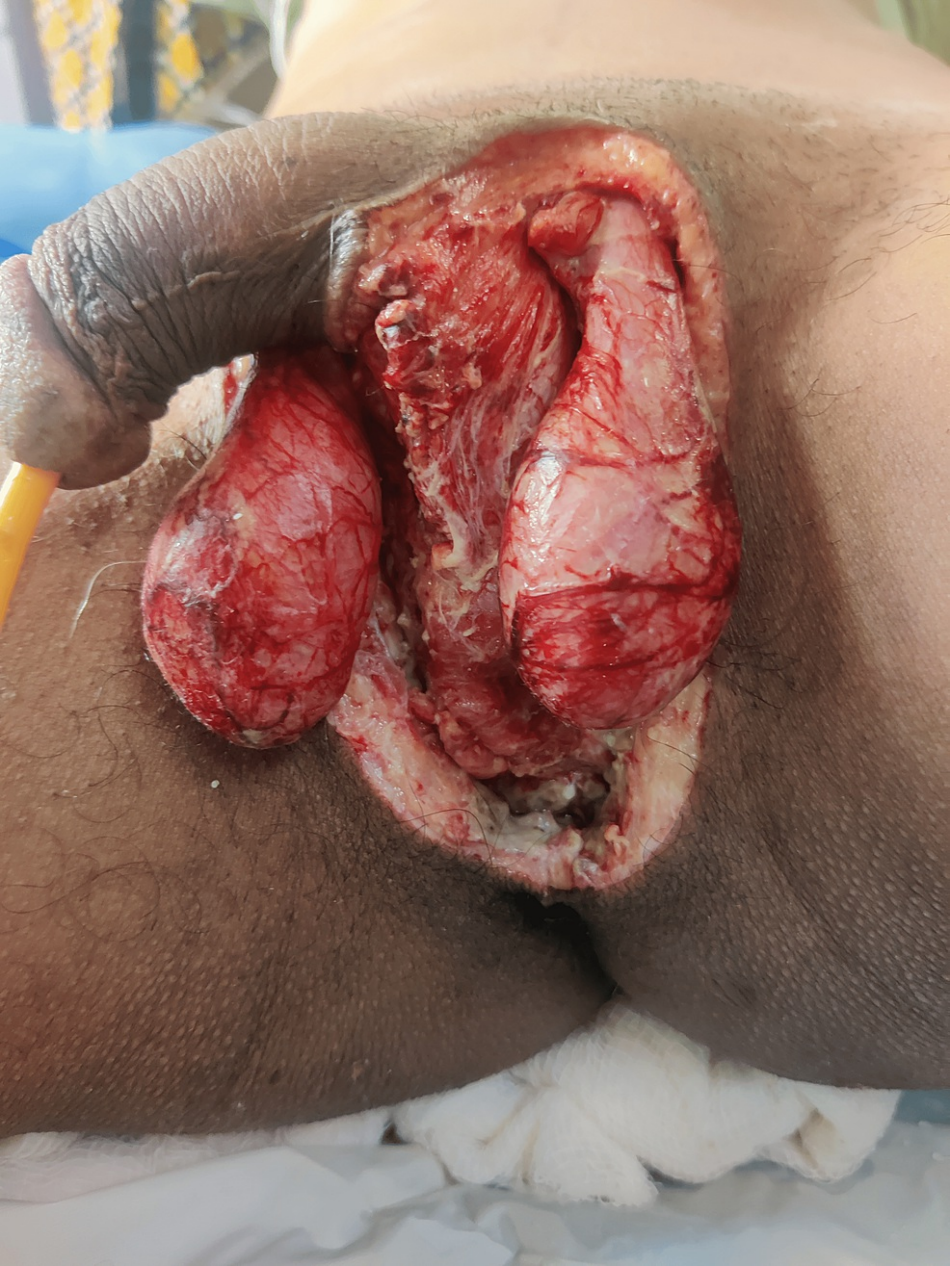
Fournier's gangrene after initial debridement

**Figure 3:**
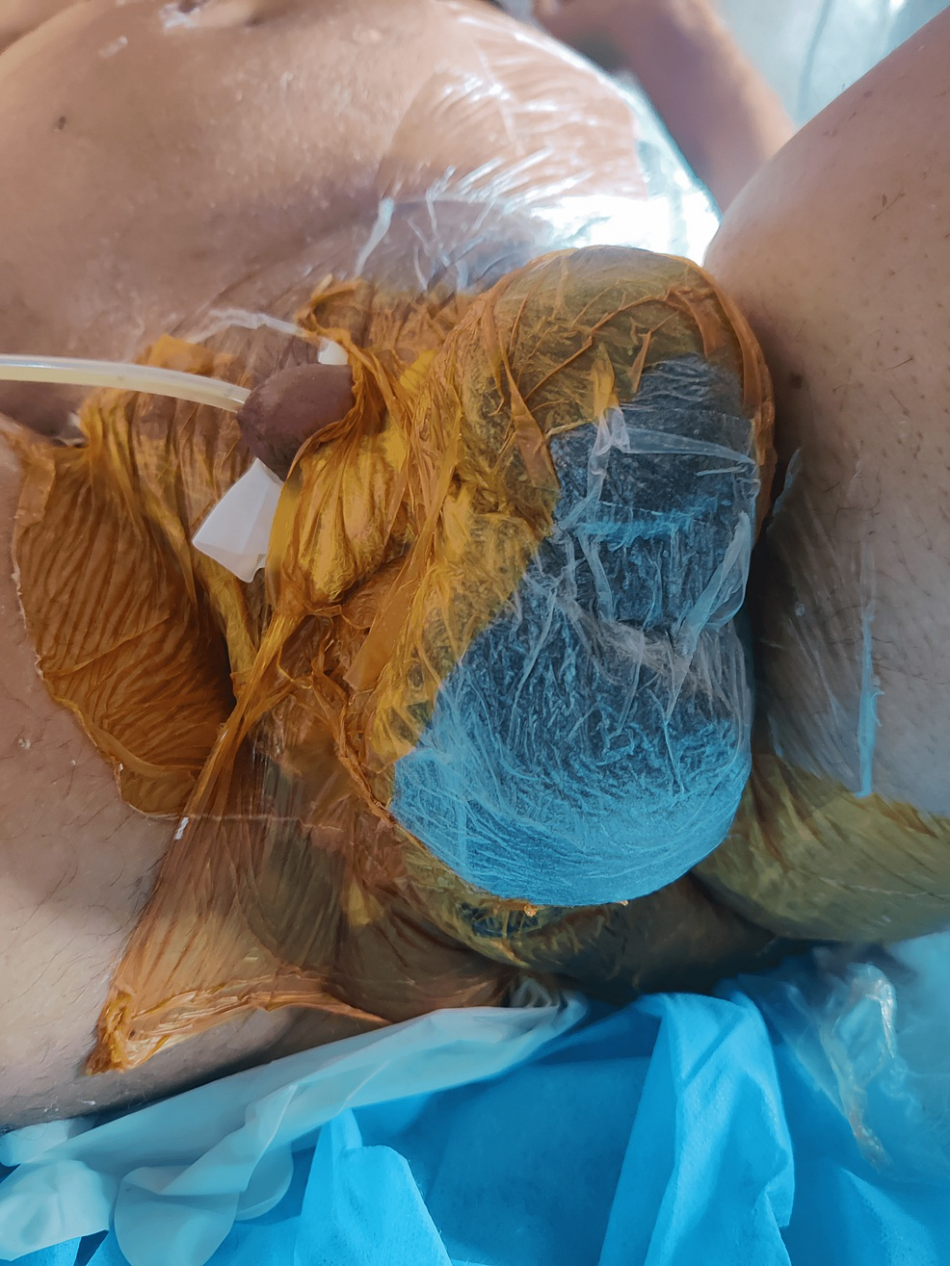
Application of vacuum-assisted closure dressing in Fournier's gangrene

**Figure 4:**
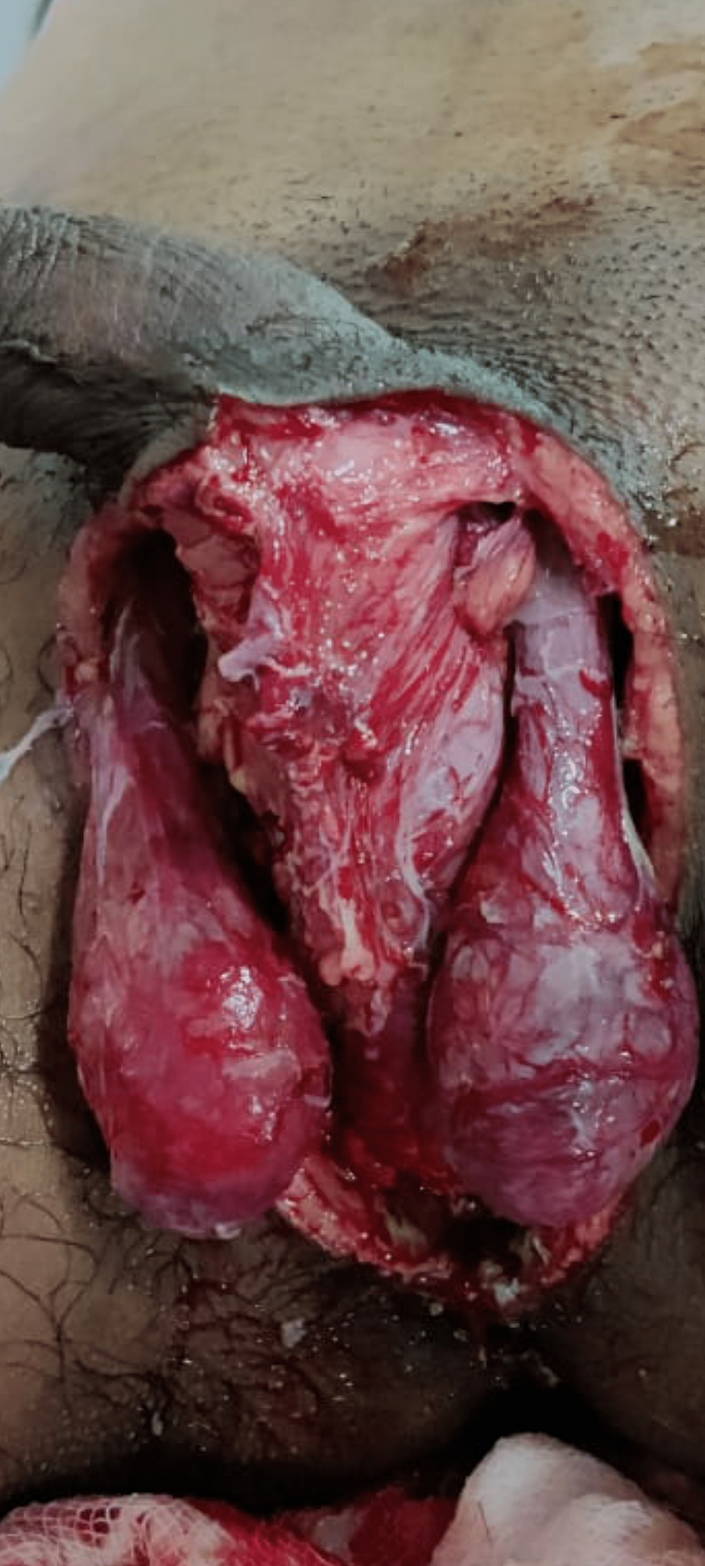
Fournier's gangrene wound after the third vacuum-assisted closure dressing

Figure 1 shows the example of a patient with FG at the time of presentation. Figure 2 shows FG after initial debridement. Figure 3 shows the application of VAC dressing in the case of FG. Figure 4 shows the results after the application of three VAC dressings over FG.

## Discussion

Iacovelli et al.'s study does not mention that VAC therapy is better than conventional dressing as far as clinical outcome is concerned [14]. However, vacuum dressing appears as an effective method in terms of fewer dressing changes, reduction in pain, and increased mobility as compared to conventional dressing. Our study on the other hand shows that VAC therapy has better clinical outcomes, as calculated by sepsis markers, FGSI scoring, and in improving patient quality of life in terms of decreased pain, increased mobility, fewer dressing changes, decreased length of hospital stay, and comparatively early wound closure, and is effective modality of treatment for FG.

The age distribution of patients in our study ranged from 22 to 81 years with a mean age of 57.48 ± 15.74 in the conventional group and 50.83 ± 13.95 in the VAC dressing group. Aridogan et al. found in their study a similar mean age of 61.3 years with a range between 36 and 92 years [15]. Thus, although FG increases with age, it can occur in any age group. While in the study conducted by Yücel et al., the mean age of the patients was 54.3 years (range: 27-82 years) [16,17].

The study done by Yanaral et al. showed that VAC application is associated with decreased time of hospital stay [18]. In the study done by Ozkan et al., the mean hospital stay period in patients who used NPWT was 18 days, while it was 20 days in the others [19]. Patients with VAC dressing were admitted to the hospital for 8.14 ± 3.13 days whereas patients with conventional dressing were admitted for 11.36 ± 4.75 days, which has a statistically significant value of 0.001. This shows that the application of VAC in FG helps in decreasing the duration of hospital stay.

Regarding the status of the wound, all patients of both the conventional and VAC dressing methods were discharged with healthy granulating wounds (photo gallery), and the wound closure was assessed on the basis of time taken for wound closure. A study done by Pastore et al. [20] also showed that VAC therapy is an effective method for wound closure as the conventional dressing method.

The average time taken for wound closure of patients in whom conventional dressing was done was 112.56 ± 13.82 days and in patients in whom VAC dressing was done was 63 ± 14.81 days, with a statistically significant p-value of <0.001. This signifies that the application of VAC therapy helps in the early closure of wounds and therefore improves patients' quality of life.

FGSI was first described by Laor et al., the mortality after the initial evaluation was a 75% probability of death with a score > 9 [21,22]. By quantifying the severity of infection using common vital signs such as temperature, heart rate, respiratory rate, and laboratory data (serum sodium, serum potassium, serum creatinine, serum bicarbonate, hematocrit, and white blood cell count), the FGSI score helps prognosticate progression and predict the mortality of the patients [23]. Vyas et al. found the FGSI mean of 10 ± 0.89 at admission was associated with increased mortality, with a threshold value of more than 8 [24]. In our study, at the time of admission, the patients in whom VAC was applied had an average score of 6, and patients in whom conventional dressing was done had an average score of 7. However, after initial debridement, the VAC dressing group had an average score of 2, and the conventional dressing group had an average score of 3, with a statistically significant p-value of 0.02. At the time of discharge, the conventional group had an average FGSI value of 2 whereas the VAC group had an average value of 0, which leads to a statistically significant p-value of 0.001. Therefore, in our study, the patients in whom VAC dressing was applied had a lesser probability of mortality than the patients in whom conventional dressing was done, as per the FGSI scoring system.

Sepsis markers

A comparison of sepsis markers was done on the basis of CRP levels, N:L ratio, procalcitonin levels, and serum albumin levels, as done by Iacovelli et al. [14].

CRP Level

At the time of admission, the average CRP value of the conventional group was 200 mg/l and the average value of the VAC group was 150 mg/l. After the initial debridement, the group in which conventional dressing was done had an average CRP value of 115 mg/l, and the average value of the VAC group was 98 mg/l, which shows a statistically significant p-value of 0.001. At the time of discharge, the conventional dressing group had an average CRP value of 70 mg/l, and the average CRP value of the VAC group was 86 mg/l, which is statistically significant with a p-value of 0.004. This means that patients in whom VAC dressing was applied had a better prognosis in terms of CRP values.

N:L Ratio

At the time of admission, the average N:L ratio of the conventional dressing group was 6.05, and of the VAC dressing group was 6.02. After debridement, patients in whom conventional dressing was done had an average N:L ratio was 6.4, and for patients in whom VAC dressing was applied was 7, which had a p-value of 0.54, which is not statistically significant. At the time of discharge, the average N:L ratio in the conventional dressing group was 6.8, and in the VAC dressing group, it was 6.55, which has a p-value of 0.96, which is not statistically significant.

Procalcitonin Levels

At the time of admission, patients in whom conventional dressing was applied had an average value of serum procalcitonin of 0.32ng/l, and patients in whom VAC dressing was applied had an average value of 0.30 ng/l. After debridement, patients in whom conventional dressing was done had an average value of 0.23 ng/l of serum procalcitonin and patients in whom VAC dressing was applied had an average value of 0.22 ng/l with a p-value of 0.80, which is not statistically significant. At the time of discharge, the average value of serum procalcitonin in the conventional dressing group was 0.20 ng/l, and the average value of serum procalcitonin in the VAC group at the time of discharge was 0.20 ng/l, but there was a statistically significant p-value of 0.004.

Serum Albumin Levels

At the time of admission, the patients in whom conventional dressing was applied had an average value of serum albumin of 2.60 g/l, and patients in whom VAC dressing was planned had an average value of 2.10. After initial debridement, patients in whom conventional dressing was done had an average value of 2.5 g/l of serum albumin and patients in whom VAC dressing was applied had an average value of 3.05 g/l, with a statistically significant p-value of <0.001. This signifies that there is less loss of serum albumin in cases of vac application, which promotes better wound healing reducing edema and adding to wound contracture.

Pain Assessment

Pain assessment scoring has been done on the basis of the numeric pain rating scale, as adopted from McCaffery and Beebe [25]. As per a study done by Ozturk et al. (2009) with the use of VAC, the patients had fewer dressing changes, less pain, fewer skipped meals, greater mobility, and reduced VAC hands-on time of the surgeons [26].

At the time of admission, the average pain assessment score in patients in whom conventional dressing was done was 9 and the group in which VAC dressing was applied was 8. However, after initial debridement, in the group in which conventional dressing was done, the average pain assessment score after dressing was 5.5, and in the group in which VAC dressing was done, the average pain assessment score was 3, with a statistically significant p-value of < 0.001. Therefore, VAC dressing application helps in reducing patient pain and hence helps in improving patient quality of life.

## Conclusions

FG occurs most commonly in the age group of 45-60 years of age, and the application of VAC therapy in FG helps in decreasing the duration of hospital stay. The application of VAC therapy helps in early wound closure in patients with FG, and patients in whom VAC dressing is applied have a lesser probability of mortality than patients in whom conventional dressing was done, as per the FGSI scoring system. Sepsis markers such as serum CRP and serum procalcitonin show a significant reduction in their value after VAC application, which is also statistically significant, although there is less loss of serum albumin in cases of VAC application, which promotes better wound healing, which is statistically supported in this study. VAC dressing application helps in reducing patient pain and hence helps in improving patient quality of life.
